# Pneumatocele formation in adult pulmonary tuberculosis during antituberculous chemotherapy: a case report

**DOI:** 10.4076/1757-1626-2-8570

**Published:** 2009-09-09

**Authors:** Liao Wan-Hsiu, Lin Sheng-Hsiang, Wu Tsu-Tuan

**Affiliations:** 1 Department of Family Medicine, Taipei County HospitalTaipei CountyTaiwan (R.O.C.); 2 Department of Internal Medicine, Taipei County HospitalTaipei CountyTaiwan (R.O.C.)

## Abstract

**Introduction:**

Pulmonary pneumatoceles are thin-walled, air-filled cysts that develop within the lung. Most often, they occur as a sequela to acute pneumonia, commonly caused by *Staphylococcus aureus*, and are found more frequently in infants and young children. Adult tuberculous pulmonary pneumatoceles are seldom reported.

**Case presentation:**

We reported a case of pulmonary tuberculosis with pneumatocele formation after antituberculous treatment. A 41-year-old man presented with fever and productive cough for 3 weeks. Chest X ray revealed cavitary lesions in bilateral upper lobes of the lung. Acid-fast rods were found in sputum and the cultures subsequently yielded *Mycobacterium tuberculosis*. After antituberculous treatment, obvious pneumatocele formation was noted in the right upper lobe.

**Conclusion:**

The formation of pneumatoceles in adult pulmonary tuberculosis can occur before, during or after antituberculous treatment, and the development of complications of pneumatoceles was variable.

## Introduction

Pulmonary pneumatoceles are thin-walled, air-filled cysts that develop within the lung. Most often, they occur as a sequela to acute pneumonia, commonly caused by *Staphylococcus aureus* and are found more frequently in infants and young children. Adult tuberculous pulmonary pneumatoceles are seldom reported. In this article, we demonstrated the formation of pneumatoceles during antituberculous chemotherapy in a 41-year-old male with pulmonary tuberculosis.

## Case presentation

**Figure 1.**
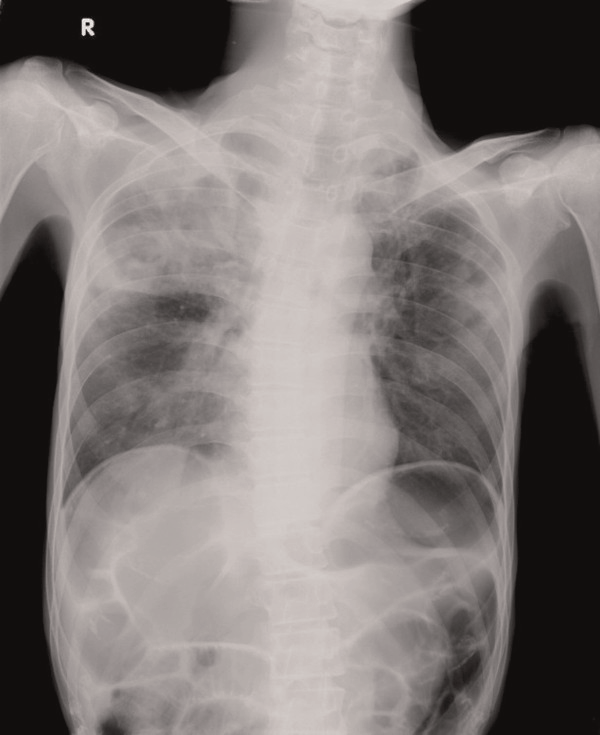
Chest radiograph at initial showing consolidation in bilateral upper lobes of the lung with multiple cavities and non-cavitary disease in the right middle lobe.

**Figure 2.**
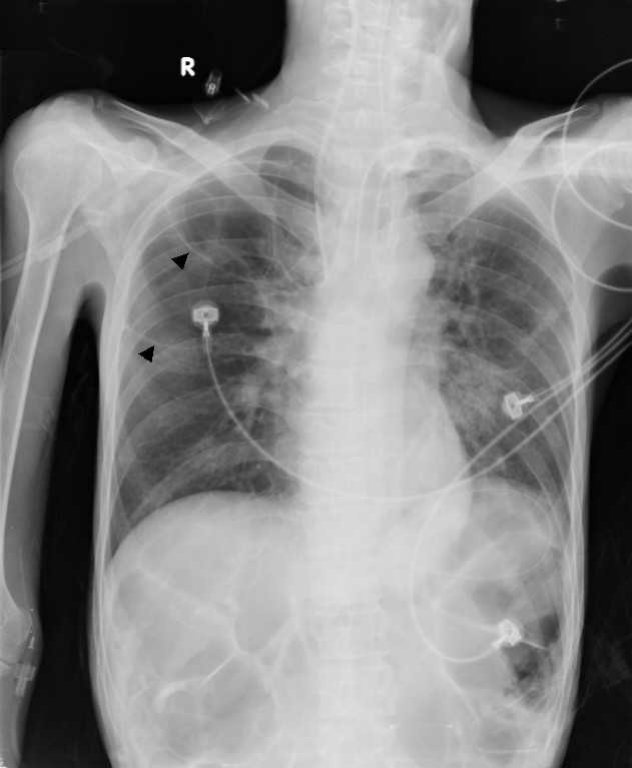
Chest radiograph at 3-month showing right upper lobe opacity replaced by multiple thin-walled cystic lesions (arrowheads) measuring greater than 4 cm in diameter.

A 41-year-old Taiwanese man presented with a 3-week history of fever, night sweats, and productive cough. The patient had cerebral palsy and activities of daily living were partially dependent. Bilateral crackles were heard on chest auscultation. His white blood cell count was 4820/mm^3^ (neutrophil 64.1%, lymphocyte 30.3%). Admission chest radiography (Figure 1) revealed consolidation in bilateral upper lobes of the lung with multiple cavities and non-cavitary disease in the right middle lobe. Acid-fast rods were found in sputum and the cultures subsequently yielded *Mycobacterium tuberculosis*. He received antituberculous chemotherapy (isoniazid, ethambutol, rifampicin and pyrazinamide for 2 months followed by isoniazid, ethambutol, and rifampicin). During the course of his illness, he was mechanically ventilated for 2 months and received tracheostomy. The patient recovered gradually and was discharged from hospital. A follow-up film 3 months later (Figure 2) showed the right upper lobe opacity had been replaced by multiple thin-walled cystic lesions (arrowheads) measuring greater than 4 cm in diameter. Since no bulla was noted in the initial images, the radiographic findings were compatible with a diagnosis of tuberculous pneumatocele. Finally, he received 9 months of antituberculous chemotherapy and there was no evidence of recurrence in one-year follow up.

## Discussion

Pulmonary pneumatoceles are air collections in the interstitium of the lung. Mostly, they occur as a sequela to acute bacterial pneumonia, reported as *Staphylococcus aureus* [1], *Streptococcus pneumoniae* [2], *Proteus mirabilis* [3], *Escherichia coli* [4], or *Acinetobacter calcoaceticus* [5]. Noninfectious etiologies include hydrocarbon ingestion, trauma, and secondary to positive pressure ventilation. Pneumatocele formation in adult pulmonary tuberculosis had been seldom reported [6,7]. In the report by Duttaroy et al. [6], tuberculous pulmonary pneumatocele communicating extrathoracically was recognized initially and the patient showed a remarkable clinical and radiological improvement after 8 weeks of antituberculous treatment. In the report by Long et al. [7], pneumatocele formation was noted following fully treated tuberculosis. In our patient, the pneumatoceles developed during antituberculous treatment without complication and our Medline research had not allowed us to identify any such cases in adult patients.

## Conclusion

Therefore, the formation of pneumatocele in adult pulmonary tuberculosis can occur before, during or after antituberculous treatment and the development of complications of pneumatoceles was variable.
